# Recognition of Ocular Artifacts in EEG Signal through a Hybrid Optimized Scheme

**DOI:** 10.1155/2022/4875399

**Published:** 2022-01-17

**Authors:** Santosh Kumar Sahoo, Sumant Kumar Mohapatra

**Affiliations:** ^1^Department of Electronics & Instrumentation Engineering, CVR College of Engineering, Hyderabad, Telangana, India; ^2^Department of Electronics and Telecommunication Engineering, Trident Academy of Technology, Bhubaneswar, Odisha, India

## Abstract

Brain computer interface (BCI) requires an online and real-time processing of EEG signals. Hence, the accuracy of the recording system is improved by nullifying the developed artifacts. The goal of this proposal is to develop a hybrid model for recognizing and minimizing ocular artifacts through an improved deep learning scheme. The discrete wavelet transform (DWT) and Pisarenko harmonic decomposition are used for decomposing the signals. Then, the features are extracted by principal component analysis (PCA) and independent component analysis (ICA) techniques. After collecting the features, an optimized deformable convolutional network (ODCN) is used for the recognition of ocular artifacts from EEG input signals. When artifacts are sensed, the moderation method is executed by applying the empirical mean curve decomposition (EMCD) followed by ODCN for noise optimization in EEG signals. Conclusively, the spotless signal is reconstructed by an application of inverse EMCD. The proposed method has achieved a higher performance than that of conventional methods, which demonstrates a better ocular artifact reduction by the proposed method.

## 1. Introduction

Electroencephalogram (EEG) signals are affected by artifacts in the recorded electrical activity; thereby, it affects the analysis of EEG. To extract clean data from EEG signals and to improve the efficiency of detection during encephalogram recordings, a developed model is required. Although various methods have been proposed for the artifacts removal process, still research on this process continues. Even if several types of artifacts from both the subject and equipment interferences are highly contaminated with the EEG signals, the most common and important type of interference is known as ocular artifacts. Electroencephalogram (EEG) is the key component in the field of analyzing brain activity and behavior. Jaffino et al. [1] proposed a grey wolf optimized-based approach for detecting epileptic seizure with an acceptable efficiency. Obukhov et al. [2] have proposed a method of feature extraction from EEG by an application of wavelet scheme. Although their model has some advantages, the performance level was not up to a satisfactory level. Sawangjai et al. [3] experimented by generative adversarial network approach for removing ocular artifact from EEG signal with a moderate sensitivity. Similarly, Peterson et al. [4] reviewed towards signal-to-noise ratio for an ITER. A model using combined methods of wavelet-ICA and SVM has proposed by author [5] to improve the elimination process of artifacts without any loss of data in EEG signals and without depending on any thresholding function. However, the performance of the system was limited to a certain range. However, this model needs a large number of features for training data sets when dealing with large datasets with more noise. An efficient technique for the removal process of artifacts from EEG signals has been explained by Selvan et al. [6], in which two adaptive filtering techniques combined like ANC for noise signal removal from the primary signal as well as reference signal and adaptive signal enhancement scheme for ANC output signal enhancement. The performance analysis based on real-time applications of this proposed model has revealed that it has efficiently removed the OAs from EEG signals. Peng et al. [7] have presented a new model to remove ocular artifacts from EEG signals, which was based on DWT and ANC. The accuracy of the proposed model was compared with the existing models in terms of simulated and measured data and used in real-time applications and portable environments since it has required only single channel sources. DWT and ANC eliminate artifacts in the low-frequency band even when the frequency is overlapping with the EEG signal. Yet, it has some processing overhead issues. Betta et al. [8] have established a novel method for removing ocular artifacts, which was an automated system to analyze rapid eye movement (REM) signals. This method has used both the detection algorithm and removal system, in which the detection algorithm has included the correlation of DWT and adaptive filtering techniques to improve the performance of artifact removal system with better accuracy. Quazi et al. [9] have implemented an algorithm for removing artifacts from EEG signals, which was based on a hybrid scheme, namely, Firefly-Levenberg-Marquardt (FLM) algorithm. The performance evaluation of the proposed model was conducted based on three factors, namely, mean square error, signal-to-noise ratio (SNR), and computational time. The estimated results have shown that the implemented model based on FLM algorithm has delivered increased performance in the process of mitigating the artifacts from EEG signals. FLM provides accurate results in removing the artifacts from EEG signal. However, this model may have a chance to fall into the local minima problem. Jafarifarmand et al. [10] have developed a model with the combination of two approaches namely, ICA and ANC. The ICA technique has been used to extract the source signals of artifacts as independent components. The extracted results have been used in the ANC technique based on neural networks. The analyzed results have shown that the developed model has offered better performance for identifying and reducing the artifacts in EEG signals. ICA-ANC gives better performance in artifact removal from EEG with the use of parallel cleaning procedure. However, it shows weak performance in following the changes during online analysis. In the medical diagnosis field, the EGG signals are used for brain electrical activity recordings. The EEG signals are often contaminated with different types of artifacts, and among them, ocular artifacts are considered as the major sources of noise. The identification and removal of ocular artifacts from EEG signals is considered as a main challenging task. The evaluation of electrical activity inside a brain is carried out by EEG using electrodes attached to the scalp. This process is known as noninvasive brain imaging technique [11, 12]. The advantages of using EEG signals in the medical field are fast functionality, safe to use, relatively inexpensive, simple to operate, and portability. On the other hand, several artifacts of technical and biological origin highly contaminate the EEG signals [13–15]. The most common types of artifacts are arising from muscle activities, heartbeat, eye blink, or movements. These artifacts are considered as a major hindrance in the analysis of EEG signals. The human eyes produce a large electric potential during eye blinks, and the resulting signal is known as Electro-Oculo-Gram (EOG). The EOG signal spreads all over the scalp, which contaminates the EEG signal that are known as ocular artifacts [16, 17]. These ocular artifacts interfere while measuring the brain signals and produce significant changes in measurements, which may induce negative waveforms with high amplitude. Therefore, the recognition and removal of ocular artifacts from EEG signals are an essential process. Various techniques are available for the removal of ocular artifacts from EEG signals [18].

In the past research works, singular value decomposition (SVD) and PCA have been used to remove ocular artifacts. Although both methods have been used for recognizing the artifacts, it has not removed it completely due to some wrong assumptions while measuring the EEG signals. Adaptive filtering is another technique that has been used for the removal of ocular artifacts. It also has some restrictions in the results due to ignorance of some information among electrodes [19, 20]. ICA is a technique that has been used for analyzing and then eliminating the ocular artifacts from EEG signals. This technique includes linear transformation, which optimizes the statistical dependence among the independent components (ICs) since the ICs lost the data in EEG signals [21, 22]. However, the ICA is not trained well for removing the ocular artifacts completely. Blind source separation (BSS) algorithm has used to separate the EOG and EEG into ICs statistically. The separation process was done again on EEGs with inverted EOG channels. However, it has some restrictions on reference EOG channels [23].

Adaptive noise cancellation (ANC) and DWT techniques are used to remove ocular artifacts from EEG signals [24–28]. This method can perform using a single EEG signal without the need of EOG signal. Although this model has given reasonable results with superior performance, it has dependent on wavelet form and threshold function, which leads to the loss of data in EEG signals [29, 30]. The existing models have a lot of challenges to overcome. Thus, deep learning is used to solve the issues in the conventional methods. It also provides various techniques, which efficiently remove ocular artifacts from the EEG signals. The followings are the advantages of using deep learning methods: (a) strong generalization ability, (b) time saving, (c) nonuse of additional EOG reference signals, etc. Most of the deep learning models provide high clearance in the process of recognizing and mitigating the ocular artifacts from EEG signals [3, 31–34]. Therefore, it is necessary to develop new methodologies to solve the abovementioned challenges and to remove the ocular artifacts efficiently. The main contributions of this paper are given as follows:

- To develop a proposed model for detection and removal of ocular artifacts from EEG-signals by various techniques like 5-level DWT and Pisarenko harmonic method for decomposition of signals, PCA and ICA for features extraction from signals, EMCD for decomposition of signals, and optimized DCN with DS-EFO for detection of ocular artifacts with enhanced accuracy rate
- Furthermore, to develop an efficient detection method of ocular artifacts using optimized DCN with the developed DS-EFO algorithm by optimizing the parameters of DCN with the aim of multiobjective function in terms of maximizing the accuracy and precision and to validate the efficiency of detection and prevention phases of ocular artifacts model by estimating with trained data sets from BCI applications by various performance metrics through comparing with the existing algorithms
- The chirplet transform is used to evaluate the performance on RMSE of the proposed scheme

## 2. Deep Learning-Based Detection and Prevention of Ocular Artifacts from EEG Signals

### 2.1. Proposed Architecture

**Figure 1:**
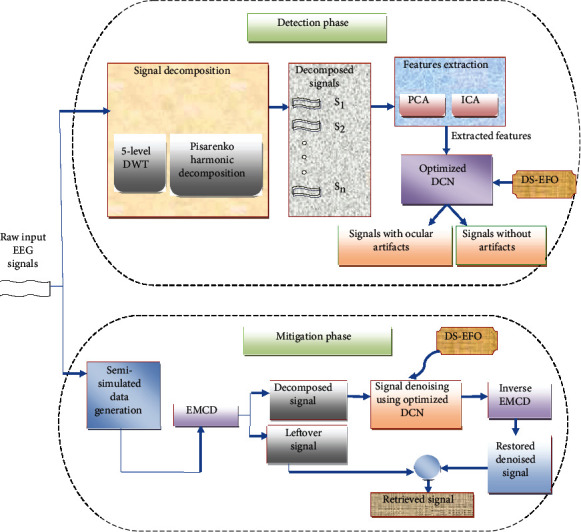
Proposed architecture for detection and mitigation of ocular artifacts from EEG signal.

In recent years, the medical field used EEG signals for several brain-related evaluations. Generally, the EEG signals have some drawbacks like diverse types of noise signals, low SNR rate, overlapping of noise and artifacts, and nonlinearity and stationary properties. Among those, artifacts are the most dangerous issue, which has the capability of degrading the efficiency of EEG signals. The artifacts in EEG signals may cause electronic saturation with high amplitude, which may affect the EEG signals and lead to provide improper results in BCI applications. Several types of artifacts can affect EEG signals in different ways. One of the most common types of artifacts is ocular artifact. The ocular artifacts are caused due to the overlapping of EOG and EEG signals in terms of both time and frequency domains. The ocular artifacts are 10 to 100 times stronger than EEG signals, which is the major drawback of ocular artifacts in EEG signals. Hence, it is considered as a challenging task to remove ocular artifacts from EEG signals. Various techniques are used to identify and remove the ocular artifacts from EEG signals like DWT, ICA, PCA, BSS, and FLM. Although these techniques provide reasonable results in the process of recognizing and removal of artifacts, it also has some limitations like does not have the ability to remove the artifacts completely with high accuracy, needs additional EOG recordings, requires multichannel EEG signals, etc. Therefore, the deep learning techniques are used in this paper to achieve accurate results with efficient diagnosis and mitigation process of ocular artifacts from EEG signals. The diagrammatic representation of the proposed detection and mitigation of ocular artifacts from EEG signal is depicted in Figure 1.

The proposed ocular artifact diagnosis model has two phases “(i) Detection phase and (ii) Mitigation phase”. The detection phase consists of three processes such as “signal decomposition, feature extraction and ocular artifacts detection”. Initially, the raw EEG input signals are decomposed by two decomposition techniques such as 5-level DWT and Pisarenko harmonic decomposition technique. The input EEG signal is decomposed into a number of samples, and the samples are examined one by one for efficient processing. Then, the decomposed signals are given as the input for PCA and ICA, in which the features are extracted from the decomposed signals. This helps to reduce the redundant features. The features extracted from the PCA and ICA are concatenated and forwarded to a deep learning technique, namely, optimized DCN, in which the epoch and learning rate are optimized using distance sorted EFO (DS-EFO). The optimized DCN is trained to classify the signals from the extracted features. Therefore, the trained optimized DCN by DS-EFO provides the output signal with artifacts and signal without artifacts. The objective function in terms of precision and accuracy ensures the efficient detection of ocular artifacts between the input and detected artifact signals.

The mitigation phase has initiated once the ocular artifacts are detected in the first phase. The mitigation phase has various steps like “signal decomposition, signal denoising and signal recovery.” The semisimulated data are generated from the signals with ocular artifacts, and it is divided into decomposed signal and leftover signal by using EMCD decomposition technique. The decomposed signal is forwarded to the optimized DCN by DS-EFO for producing the denoised signals, which is further processed through inverse EMCD to generate artifact restored denoised signal. Then, the artifacts removed signals or retrieved signals are generated by summing the leftover signals and the restored denoised signal. Here, the objective function lifting the efficiency of mitigation of ocular artifacts between the clean signal and retrieved signal is to reduce the MAE between them.

### 2.2. Signal Decomposition Phase

The initial step of efficient signal processing is signal decomposition, in which the signal components are extracted and separated into a greater number of samples. The first phase of decomposition of the input EEG signal *S*_*n*_ is done by 5-level DWT. The collected input EEG signals are termed as *S*_*n*_, where *n* = 1, 2, ⋯, *N* and *N* represent the total number of input EEG signals.

Discrete wavelet transforms (DWT) [28]: it is a wavelet transform technique that decomposes the input EEG signals into a number of samples, where each sample is a time series of coefficients. The coefficients describe the signal evolution time related to the frequency bands. The frequency of the signal is divided into low and high frequency bands by DWT. The low frequency band is further divided into low and high frequency phases. The high frequency band contains the data of the edge and surface of the signal. In the 5-level DWT decomposition method, the level 1 decomposition of the signal produces four number of subfrequency bands like LFLF1, LFHF1, HFLF1, and HFHF1. The LFLF1 subfrequency band in the top level is given as input for the next level decomposition. The decomposition process of the remaining levels is as follows:

- For the decomposition of level 2, the DWT is employed on LFLF1 subband, which is the previous level. The level 2 decomposition generates four subfrequency bands such as LFLF2, LFHF2, HFLF2, and HFHF2
- Likewise, the level 3 decomposition produces 4 subbands such as LFLF3, LFHF3, HFLF3, and HFHF3 by applying the DWT to the LFLF2, i.e., level 2
- For the level 4 decomposition, the DWT is applied to level 3, i.e., LFLF3 band. Therefore, the level 4 decomposition delivers four subfrequency bands such as LFLF4, LFHF4, HFLF4, and HFHF4
- Finally, the decomposition of level 4 is done by applying DWT to the LFLF4 band. The level 4 generates four subfrequency bands like LFLF5, LFHF5, HFLF5, and HFHF5

The signal is transmitted to the filter series for the measurement of DWT of a signal *sdt*. Initially, the samples are transmitted through a low pass filter with impulse response *flp*.The result is generated as shown in Eq. (1).

(1) $$\begin{array}{c} F\left[g\right]=\left(\text{sdt}\times \text{flp}\right)\left[g\right]=\overset{\infty }{\underset{n=-\infty }{\sum }}\text{s}\text{d}t\left[m\right]\text{flp}\left[g-m\right]. \end{array}$$

Similarly, the high pass filter is *flp* is also used for signal decomposition. The output of low-pass filter is resampled by 2. Thus, the signal is again transferred to a new “low-pass filter and high-pass filter” for further processing by half the cut-off frequency of the final one. The process is defined in the formulas of Eq. (2) and Eq. (3).

(2) $$\begin{array}{c} F_{\text{low}}\left[g\right]=\overset{\infty }{\underset{\backslash n=-\infty }{\sum }}\text{s}\text{dt}\left[m\right]\text{flp}\left[2g-m\right], \end{array}$$

(3) $$\begin{array}{c} F_{\text{high}}\left[g\right]=\overset{\infty }{\underset{n=-\infty }{\sum }}\text{s}\text{dt}\left[m\right]\text{fhp}\left[2g-m\right]. \end{array}$$

Hence, the decomposed signal *S*_*n*_^DWT^ is generated by using DWT technique.

Pisarenko harmonic decomposition [17]: the next phase decomposition of *S*_*n*_^DWT^ is accomplished by Pisarenko harmonic decomposition technique. Generally, this technique is familiar for frequency estimation, in which the eigenvector *e*^*jk*^ corresponding to the lowest eigenvalue *e*^*H*^ of the input signal is used for evaluation, and the result is generated as shown in Eq. (4).

(4) $$\begin{array}{c} {\overset{⌢}{P}}_{\text{pkh}}\left(e^{jk}\right)=\frac{1}{{\left|e^{H}{nv}_{\min}\right|}^{2}}. \end{array}$$

Here, the term *nv*_min_ refers to the noise eigenvector, where *e* = [1*e*^*jk*^*e*^*j*2*k*^ ⋯ *e*^*j*(*M* − 1)*k*^]^*T*^. Thus, the decomposed signal *S*_*n*_^PH^ is generated by using Pisarenko harmonic decomposition technique.

### 2.3. Proposed DS-EFO

The detection and mitigation of ocular artifacts is effectively improved by a developed heuristic EFO algorithm, namely, DS-EFO. The DCN technique is used to detect and mitigate the artifacts in the signals. The parameters of DCN such as epoch and learning rate are optimized by DS-EFO algorithm to improve the efficiency of detection. EFO algorithm is based on swarm intelligence. This algorithm has done many optimizations on the swarm intelligence, and it is a better algorithm to solve some complex problems. However, it needs more steps for solving the problems thereby it takes much time for computation. Therefore, the DS-EFO is proposed to overcome the limitations of the existing EFO by simplifying the process, thereby reducing the computation time.

EFO [29]: EFO is simulated based on the communication behaviors of electric fish, namely, nocturnal electric fish. Generally, this electric fish lives in a muddy water surfaces, where the visual capacity of electric fish is narrow. This electric fish with poor eyesight depends on their species-specific ability known as electrolocation to recognize the environment. Electrolocation refers to the sense of the ability of the electric fish to differentiate between prey and obstacles. There is an electric organ in the electric fish, which has disc-like-cells called electrocytes. This organ is located at the tail of an electric fish, and it is used to generate an electric field. Electric organ discharge (EOD) is generated due to the simultaneous excitation of these electrolytes. EOD is identified by its amplitude and frequency. The amplitude of electric field finds the effective range of the EOD in local search, and this parameter is depending on the size of the fish. The electric fish which are closest to the optimal source generates high frequency of electric field, and the time corresponding to the frequency tf is measured for everyone. Electrolocation is categorized into active and passive based on the capability of the fish in searching and locating the prey. The active electrolocation has a limited range, and the electric fish can be able to sense the near areas to identify their prey and generate EOD through the changes in the electric field. On the other hand, the passive electrolocation has a wider range than the active electro location, which leads the electric fish to find the location of a distanced objects and able to communicate with other fish. Thus, to find the best food source quality from the infinite food source of everyone with the time frequency tf in the large dimensional search space, the computational steps of EFO algorithm are formulated in the following equations.

In the conventional EFO algorithm, the solutions are updated based on different constraints and that leads to computational and time complexity. Therefore, the proposed DS-EFO algorithm is introduced based on the distance among the solutions. It is executed by only one constraint called distance, which makes the algorithm as a simpler one. Here, the distance is computed between the best solution and the current solution. Then, the mean of distance is computed. If the distance of the current solution is lesser than the mean distance and there exists at least one neighbor in the active sense area, then the solutions are updated based on active electrolocation. If the condition fails, then the solutions are updated based on passive electrolocation.

Population initialization: the collection of individuals or electric fish population is spreading in the search space in a random manner. The population initialization with the determination of boundaries is formulated in Eq. (5).

(5) $$\begin{array}{c} {xz}_{pq}={xz}_{\min q}+\delta \left({xz}_{mzxq}-{xz}_{\min q}\right). \end{array}$$

Here, the term *xz*_*pq*_ refers to the location of the individual *p* in the dimensional search space with the population of size |NP|, where *p* = 1, 2 ⋯ (|NP|). The term *δ* denotes the uniform distribution. The lower and upper boundaries of the search space are indicated by *xz*_min*q*_ and *xz*_max*q*_, respectively.

After the population initialization process, the probability of individuals' frequency range fr_*p*_^tf^ is determined using the minimum frequency fr_min_ and maximum frequency fr_max_ range of individuals from its fitness value. The individuals with higher frequency range use active electrolocation, and others employ passive electrolocation. The frequency value of individuals from its fitness value is formulated in Eq. (6).

(6) $$\begin{array}{c} {\text{fr}}_{p}^{\text{tf}}={\text{fr}}_{\min}+\left(\frac{{\text{fr}}_{\text{worst}}^{\text{tf}}‐\text{f}{\text{r}}_{p}^{\text{tf}}}{{\text{fr}}_{\text{worst}}^{\text{tf}}‐{\text{f}}_{\text{best}}^{\text{tf}}}\right)\left({\text{fr}}_{\max}-f_{\min}\right). \end{array}$$

Here, the terms fr_best_^tf^ and fr_worst_^tf^ denote the best and worst fitness value of individuals for the corresponding individual population at iteration tf. The probability calculation is done by using the frequency value of fr_min_ and fr_max_ which is given in the range of 0 and 1, respectively. Next, the amplitude value of the individual amp_*p*_ is calculated by the weight of the previous amplitudes *β* of individuals due to its dependence. The amplitude value depends on other passively electro locating fish, and the electric field strength decreases with the inverse cube of distance. The calculation of the amplitude value is formulated in Eq. (7).

(7) $$\begin{array}{c} {\text{amp}}_{p}^{t\text{f}}=\beta {\text{amp}}_{p}^{\text{tf}-1}+\left(1-\beta \right){\text{fr}}_{p}^{\text{tf}}, \end{array}$$

Active electrolocation: the characteristics of active electrolocation determine the exploitation capability. The amplitude value amp_*p*_ determines the active range of the individual *ar*_*p*_, and it is formulated in Eq. (8).

(8) $$\begin{array}{c} {\text{ar}}_{p}=\left({xz}_{\text{mzx}q}-x_{\min q}\right){\text{amp}}_{p}. \end{array}$$

After the calculation of an active range, the distance among the individuals *p* and the remaining population is measured. The Cartesian distance calculation is used to determine the individuals *p* and neighboring individuals kn, and it is formulated in Eq. (9).

(9) $$\begin{array}{c} {\text{dis}}_{p\text{ kn}}=\left\|{xz}_{p}-{xz}_{kn}\right\|=\sqrt{\overset{\text{dis}}{\underset{q=1}{\sum }}{\left({xz}_{p\;q}-{xz}_{\text{kn}q}\right)}^{2}}. \end{array}$$

The EFO algorithm uses the Eq. (10) formula when at least one neighbor exists in the active region.

(10) $$\begin{array}{c} {xz}_{pq}^{nand}={xz}_{pq}+\delta {ar}_{p} \end{array}$$

Passive electrolocation: the exploration capability is based on the characteristics of passive electrolocation. The probability of the individual *p* in the active mode (i.e., *p* ∈ *NP*_*a*_) being perceived by the individual kn in passive mode (i.e., nk ∈ NP_nk_) is calculated using Eq. (11).

(11) $$\begin{array}{c} {\text{ab}}_{p}=\frac{{\text{amp}}_{p}/{dis}_{p\;kn}}{\sum_{q\in {\text{NP}}_{a}}{\text{amp}}_{q}/{\text{dis}}_{pq}}. \end{array}$$

Using Eq. (11) the individuals NK selected from NP_amp_ to determine a reference location in Eq. (12), the new location *xyz*_*pq*_ is generated in Eq. (13).

(12) $$\begin{array}{c} {xyz}_{pq}=\frac{\sum_{\text{nk}=1}^{\text{NK}}{\text{amp}}_{\text{nk}}{xz}_{\text{nk }q}}{\sum_{\text{nk}=1}^{\text{NK}}{\text{amp}}_{\text{nk}}}, \end{array}$$

(13) $$\begin{array}{c} {xz}_{pq}^{\text{new}}=x_{pq}+\delta \left({xyz}_{pq}-{xz}_{pq}\right). \end{array}$$

Finally, the probability of the new location is increased by modifying a parameter of the individual *p* and it is formulated in Eq. (14).

(14) $$\begin{array}{c} \begin{array}{c} {xz}_{pq}^{nana}={xz}_{\min q}+\delta \left({xzm}_{zxq}-{xz}_{\min q}\right)\\ nand\left(0,1\right)\leq \left(0,1\right) \end{array} \end{array}$$

In the EFO algorithm, the calculation of active and passive electrolocation takes several steps to find the distance between the individuals and the location of the best food source in the given search space. In the proposed algorithm, Eqs. (10) and (14) are modified to reduce the time complexity and the computation time. The pseudocode of the proposed DS-EFO algorithm is represented in Algorithm 1.

**Figure 2:**
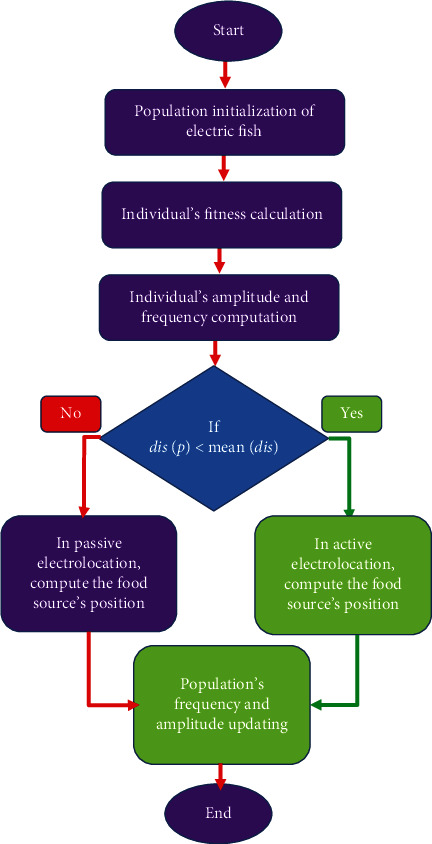
Flowchart of the proposed DS-EFO algorithm.

The flowchart of the proposed DS-EFO algorithm is represented in Figure 2.

## 3. Ocular Artifacts Detection by Optimized Deformable Convolutional Networks

### 3.1. Feature Extraction by PCA and ICA

The feature extraction process refers to transforming the input signals into numerical features for preserving the information of input data while processing. The results obtained are better while performing detection or classification tasks using the extracted features than applying to the raw input data. The features of the decomposed signal *S*_*n*_^PH^ are extracted by two analytical component techniques such as PCA and ICA.

PCA [12]: it is considered as a data reduction technique, and it uses linear algebra for feature extraction, which transforms the input data signal *S*_*n*_^PH^ into a compressed form, i.e., a small number of relevant features. The features are converted into matrix, the feature extraction process is done by evaluating the mean variables mv, and it is formulated in Eq. (15). The term *y*_*ij*_ denotes the weight, and *j* is a variable, where *j* = 1, 2 ⋯ *m* and *p* are another variable, where *p* = 1, 2 ⋯ *n*.

(15) $$\begin{array}{c} {\bar{y}}_{j}=\frac{1}{p}\overset{p}{\underset{i=1}{\sum }}y_{ij}. \end{array}$$

In Eq. (15), the term *y*_*ij*_ denotes the weight, and *j* is a variable, where *j* = 1, 2 ⋯ *m* and *p* are another variable, where *p* = 1, 2 ⋯ *n*.

(16) $$\begin{array}{c} {a^{2}}_{jj}=\frac{1}{p}\overset{p}{\underset{i=1}{\sum }}\left(x_{ij}-{\bar{x}}_{j}\right). \end{array}$$

In Eq. (16), the term *A* = {*a*_*ij*_} refers to the covariance matrix. The term *a*^2^*jj* denotes the variance. The covariance is formulated in Eq. (17).

(17) $$\begin{array}{c} a_{kj}=\frac{1}{p}\overset{p}{\underset{i=1}{\sum }}\left(x_{kj}-{\bar{x}}_{k}\right)\left(x_{kj}-{\bar{x}}_{j}\right). \end{array}$$

In Eq. (17), the variable is represented by *k* = 1, 2 ⋯ *m*. An eigenvalue *e*^*H*^ and eigenvector *e*^*jk*^ can be calculated by *Ae*^*jk*^ = *e*^*H*^*e*^*jk*^. If *A* is a *n* × *n* matrix of full rank, *n* eigenvalues and all corresponding eigenvectors are measured in Eq. (18).

(18) $$\begin{array}{c} \left(A-e^{H}I\right)e^{jk}=0. \end{array}$$

The features of decomposed signals are represented as, Fe_fs_^PCA^ where fs = 1, 2 ⋯ FS and FS denote the total number of features extracted from PCA, which are attained 83 features.

ICA [20]: it is a method for extracting features from the input signal S_*n*_^PH^, which is a multivariate random signal that has transformed into independent components. Each component carries information that will not infer to others. Numerically, the probability of each component is obtained from the feature extraction process. The multivariate density function is measured by gathering the independent components *c* into vector *z*(*t*) by assuming the vector with zero mean and the result generated as shown in Eq. (19).

(19) $$\begin{array}{c} \text{PF}\left(z\left(t\right)\right)=\overset{c}{\underset{r=1}{\prod }}\text{P}\text{F}\left(z_{r}\left(t\right)\right), \end{array}$$

(20) $$\begin{array}{c} xy\left(t\right)=Az\left(t\right). \end{array}$$

The above Eq. (20) formulates the dimensional data for each component. The main aim of ICA is recovering the source signals from the sensed signals, and it is formulated in Eq. (21).

(21) $$\begin{array}{c} xz\left(t\right)=\text{MT}xy\left(t\right)=\text{MTAz}\left(t\right). \end{array}$$

Here, the term *z*(*t*) denotes the source, and the term *xz*(*t*) indicates the estimation of *z*(*t*) and MT = *A*^‐1^. The features of decomposed signals using ICA are represented as Fe_fs_^ICA^, which is attained as 83 features. Thus, the extracted features from PCA and ICA are concatenated as Efs_fs_^ex^ = {Fe_fs_^PCA^, Fe_fs_^ICA^}, where fs = 1, 2 ⋯ FS and FS denote the total number of concatenated features.

Optimized DCN-based detection process: the efficient ocular artifact detection is performed by DCN, which is further improved by optimizing the epoch *E*_ch_ and learning rate Le_rt_ of DCN using the proposed DS-EFO algorithm. The extracted features from PCA and ICA Efs_fs_^ex^ are given as input to the optimized DCN. The optimized DCN classifies the signals with or without ocular artifacts.

DCN [7, 26]: the deformable network is established to overcome the performance limitations of the existing CNN. The DCN network has a learnable and deformable convolution and pooling layer. The deformable convolution adds offsets to the regular grid sampling locations in the standard convolution to deform the constant receptive field of the previous activation unit. Likewise, the deformable pooling adds an offset to each position in standard pooling. The preceding feature map is used to extract the offsets.

Deformable convolution: the convolution layer is the key component of CNN, which is used for extracting feature maps from the input. The two steps of regular convolution are sampling and summation. The sampling is done on the input feature map by adding the offsets to the locations in the regular convolution, and the summation is processed by using weighted kernel values. The process of feature extraction is enhanced by generating deformed sampling locations for the existing convolutions. It is modified by adding 2 modules prior to regular convolution, in which one is used to produce an offset field, and the other is used to generate deformable feature maps. The offset fields of the instantaneous value of the input signal through convolution are calculated, and the information of neighboring instantaneous values is fused to generate the deformable signal. The extracted features Efs_fs_^ex^ are given as input to the DCN. The sampling locations are shifted to neighboring locations by training the offsets field, which is generated using the weights of the convolution layer. The output generated from the deformable features using regular convolution is formulated in Eq. (22).

(22) $$\begin{array}{c} \text{cy}\left({\text{it}}_{\text{nt}}\right)=\overset{\text{NT}}{\underset{\text{it}=1}{\sum }}{\text{wc}}_{\text{it}}{\text{Efs}}_{\text{fs}}^{\text{ex}}\left(\text{it}\right). \end{array}$$

Here, wc_it_ is the kernel weight. The deformable convolution considers fractional data locations and the interneuron positions, which is not considered in regular convolution. Moreover, the deformable convolution has no fixed shape.

**Figure 3:**
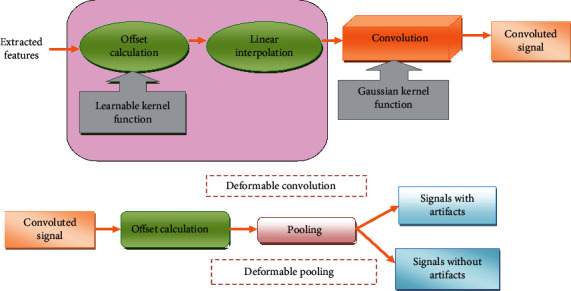
Functions of deformable convolution and deformable pooling.

Deformable pooling: in conventional pooling, the downsampling is used to minimize the size of input values to speed up the learning process. The fixed sampling locations and less efficiency of the learnable process are the drawbacks of existing convolution. The limitations of both methods are solved by the deformable region-of-interest (RoI) pooling. Before pooling, the offsets are added to the spatial positions, and the kernel weights of the downsampling are trained well by using the deformable sampling locations. The functions of deformable convolution and deformable pooling are depicted in Figure 3.

DCN consists of a deformable pooling layer preceded by a deformable convolution layer. The deformable signal is generated by applying the linear interpolation method to the input signal. The deformable signal is further given to the convolution. The calculation of trainable offset is performed on both pooling and convolution layers.

Deformable convolution layer: in the existing convolution methods, the output feature cy for each time instant it_0_ is defined as shown in Eq. (23).

(23) $$\begin{array}{c} \text{cy}\left({\text{it}}_{0}\right)=\underset{{\text{it}}_{\text{nt}}}{\sum }\text{w}\text{c}\left({\text{it}}_{\text{nt}}\right){\text{Efs}}_{\text{fs}}^{\text{ex}}\left({\text{it}}_{0}+\text{i}{\text{t}}_{\text{nt}}\right). \end{array}$$

Here, it denotes the time instants of the sampling grid SG. The regular grid SG is attached with offsetsΔit_nt_ | nt = 1, 2 ⋯ TN.

(24) $$\begin{array}{c} \text{cy}\left({\text{it}}_{0}\right)=\underset{{\text{it}}_{\text{nt}}}{\sum }\text{w}\text{c}\left({\text{it}}_{\text{nt}}\right){\text{Efs}}_{\text{fs}}^{\text{ex}}\left({\text{it}}_{0}+\text{i}{\text{t}}_{\text{nt}}+\delta \text{i}{\text{t}}_{\text{nt}}\right). \end{array}$$

In Eq. (24), the term it_nt_ + *δ*it_nt_ refers to indicate the changeable sampling locations. The term *δ*it_nt_ is typically fractional, and the linear interpolation method is used to find the new location. The equation is denoted by Eq. (25).

(25) $$\begin{array}{c} {\text{Efs}}_{\text{fs}}^{\text{ex}}\left(\text{it}\right)=\underset{v}{\sum }\text{G}\text{S}\left(v,\text{it}\right){\text{Efs}}_{\text{fs}}^{\text{ex}}\left(v\right). \end{array}$$

Here, the fractional location is denoted by it and it = it_0_ + it_nt_ + *δ*it_nt_, the term *v* denotes the spatial locations in the feature map *Efs*_*fs*_^*ex*^, the linear interpolation kernel is indicated by GS(*v*, it), and it is represented in Eq. (26).

(26) $$\begin{array}{c} \text{GS}\left(v,\text{it}\right)=\max\left(0,1-\left|v-\text{it}\right|\right). \end{array}$$

Therefore, the computation time is reduced by using deformable convolution when compared to regular convolution. Additionally, the kernels in the convolution layer as well as the offsets are learned efficiently while training. The regular sampling method of convolution layer is replaced with adaptive sampling to achieve enhanced learning.

Deformable pooling layer: this layer uses spatial pooling, which concatenates the neighboring locations and generates a summation of the joint distribution of the features. As already known that the existing pooling models are not trained and their sampling locations are fixed, the RoI pooling is used in the deformable pooling layer. The generated output is as shown in Eq. (27).

(27) $$\begin{array}{c} \text{cz}\left(\text{it}\right)=\frac{\sum_{{\text{it}}_{\text{nt}}}\text{c}\text{y}\left({\text{it}}_{0}+\text{i}{\text{t}}_{\text{nt}}+\delta \text{i}{\text{t}}_{\text{nt}}\right)}{\text{nt}}. \end{array}$$

Fully connected layer: this layer is used to determine each and every class of signal, and it is shown in Eq. (28).

(28) $$\begin{array}{c} \text{c}\hat{\text{f}}=\sigma \left({\text{czwc}}_{\text{fcl}}+\text{b}{\text{s}}_{\text{fcl}}\right). \end{array}$$

**Figure 4:**
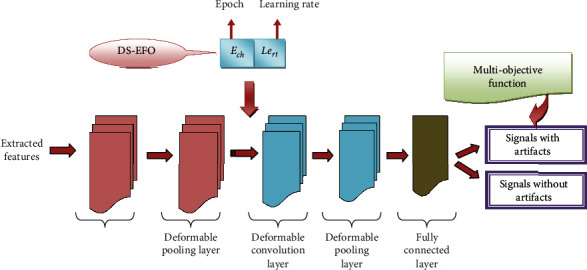
Overall architecture of DCN.

Here, the term wc_fcl_ and bs_fcl_ denotes the weight vector and bias in the fully connected layer, and the term *σ* indicates the signum function. The overall architecture of the DCN is represented in Figure 4.

The detection of ocular artifacts by optimized DCN generates the output signal with ocular artifacts or without artifacts from the raw input EEG signals. In the detection process, the optimized DCN is trained by assigning the input as extracted features Efs_fs_^ex^ and the target with the presence of ocular artifacts or not. This trained optimized DCN efficiently detects the ocular artifacts with concerning accuracy and precision.

## 4. Prevention of Ocular Artifacts by EMCD and Optimized Deformable Convolutional Networks

### 4.1. Semisimulation Data Generation

The prevention or mitigation phase of ocular artifacts from EEG signals uses the same architecture model of DCN as in the detection phase. Here, the optimized DCN is used in the process of denoising the signals. The detected ocular artifacts from the detection phase are further removed or prevented in the mitigation phase. However, there is no proof for the complete removal of ocular artifact from the signals. The semisimulated data generation is required to validate the removal process. The signals are added with some ocular artifacts are combined along with the signals with no artifacts. The signals without adding artifacts are considered as the target signal SM_*n*_^cln^ and the signals after removing ocular artifacts are assigned as the denoised signal SM_*n*_^dnoise^.

The 22 EEG and 3 EOG signals of 25 channel signals are from BCI competition IV dataset. Then, it was segmented and reshaped to 288 epochs of length 6 s (250 Hz × 6 s = 1500 time points) and data tensor, *X* ∈ *R*^25×1500×288^ respectively [35]. Furthermore, the labeled data of whether the artifact-contaminated epochs or cleaned epochs are obtained from BCI competition. Additionally, the epochs with contaminated artifacts are reshaped into matrix XY_EEGsig+Arfatcs_ ∈ *R*^25×(1500*N*_epoch_)^. Here, the contaminated epochs are denoted as*N*_epoch_.

### 4.2. EMCD-Based Signal Decomposition

The signal decomposition process involves the extraction of samples from the signal components. In the mitigation phase, the EMCD decomposition technique is used to decompose the artifactual signals into decomposed signals and left-over signals.

EMCD [36]: the decomposition algorithm of EMCD calculates the superior and inferior envelope of signal decomposition in every process. In this process, the mean curve is extracted by optimizing the envelopes by averaging it using the scale control algorithm. This EMCD algorithm uses data-driven approach, and the time series are decomposed in multiscale level. Initially, the maxima and minima are extracted from the input time series. Then, the inferior and superior envelopes are generated by using local scale control technique. The mean curve output is calculated by averaging both envelopes.

Consider SM_*n*_^af^ as {fx(*t*), *t* = 1, ⋯, *T*} where *T* is an element and the time series is referred by *f*(*t*). The minima series of fx(*t*) is denoted as {(kb_*i*_, fx[kb_*i*_]), *i* = 1, ⋯, *T*_kb_}. The time index is indicated by kb_*i*_, and the number of minima is termed as *T*_kb_. The maxima series of *fx*(*t*) is denoted as {(ma_*i*_, fx[ma_*i*_]), *i* = 1, ⋯, *T*_ma_}, in which the term ma_*i*_ indicates the time index and *T*_ma_ denotes the number of maximums. The term *W*{(*fx*_*i*_, *lx*_*i*_), *fx*_0_} is the mostly utilized B-spline interpolation function that interpolates the input series (fx_*i*_, lx_*i*_) at time point fx_0_.

Superior envelope: the upper trend curve is referred to as the time series of this envelope that passes through all of its maxima. The maxima are interpolated by applying the B-spline interpolation. The superior envelope is mathematically represented [37] in Eq. (29).

(29) $$\begin{array}{c} {\text{fx}}^{\sup}\left[t\right]=W\left\{\left({\text{ma}}_{i},\text{fx}\left[{\text{ma}}_{i}\right],\text{fx}\left[t\right]\right)\right\},t=1,\cdots ,T. \end{array}$$

Inferior envelope: the inferior envelope of a time series is the lower trend curve that passes through all of its minima. The B-spline interpolation is used to interpolate the minima. The mathematical representation of the inferior envelope is represented in Eq. (30).

(30) $$\begin{array}{c} {\text{fx}}^{\inf}\left[t\right]=W\left\{\left({\text{kb}}_{i},\text{fx}\left[{\text{kb}}_{i}\right],\text{fx}\left[t\right]\right)\right\},t=1,\cdots ,T. \end{array}$$

Mean curve: the mean curve of the time series is the average of its inferior and superior envelopes, and it represents the global trend as shown in Eq. (33).

(31) $$\begin{array}{c} {\text{fx}}^{\text{mean}}\left[t\right]=\frac{\left({\text{fx}}^{\sup}\left[t\right]+{\text{fx}}^{\inf}\left[t\right]\right)}{2},t=1,\cdots ,T. \end{array}$$

Mode: the mode of a time series is the average of the number of its maxima *T*_ma_ and that of its minima *T*_kb_.The equation of the mean curve is formulated in Eq. (32).

(32) $$\begin{array}{c} \text{md}\left(\text{fx}\left[t\right]\right)=\left(\frac{T_{\text{ma}}+T_{\text{kb}}}{2}\right). \end{array}$$

Empirical waveform: in reality, the mean curve is defined by the extrema that generate a new method to model the time series. The new concept EWF is introduced, which is a series of alternating maxima and minima. The empirical waveform is mathematically represented in Eq. (33).

(33) $$\begin{array}{c} \text{empw}\left(\text{fx}\left[t\right]\right)=\left\{\left({\text{ma}}_{i},\text{fx}\left[{\text{ma}}_{i}\right]\right),\left({\text{kb}}_{i},\text{fx}\left[{\text{kb}}_{i}\right]\right)\right\}. \end{array}$$

EWF is used to represent the mean curve when the mode of md(fx[*t*]), which characterizes this EWF. In particular, one entire sine wave cycle has one maximum and one minimum that contribute exactly one to its mode. Therefore, the EWF mode behaves like the count of entire cycles in classical Fourier analysis. Eq. (34) represents the empirical period mentioned EWF.

(34) $$\begin{array}{c} {\text{EP}}_{\text{EWF}}=\frac{T}{\text{md}\left(\text{fx}\left[t\right]\right)}. \end{array}$$

The equation for empirical frequency is represented in Eq. (35).

(35) $$\begin{array}{c} {\text{FP}}_{\text{EWF}}=\frac{\text{md}\left(\text{fx}\left[t\right]\right)}{T}. \end{array}$$

It should be noted that both the empirical period and empirical frequency are temporal evaluations over the complete time series than the original model parameters as in the conventional Fourier analysis. These conditions improve the descriptive capabilities of the signals in an efficient way that a broad signal class from oscillatory sources is designed, for example, brain regions, and neurons, and those signals are similar, but not like sine waves. Hence, the Fourier analysis decomposes this type of time series into a collection of sine waves at various frequencies, and the wavelet transform decomposes them into a set of wavelets at a number of frequencies and distinct temporal locations.

Therefore, the signals with detected ocular artifacts SM_*n*_^af^, and the semisimulated data are processed through EMCD, in which the signals are decomposed into decomposed signals SM_*n*_^decomp^ and leftover signals SM_*n*_^lefto^, and it is formulated in Eq. (36).

(36) $$\begin{array}{c} {\text{SM}}_{n}^{\text{emp}}=\left\{{\text{SM}}_{n}^{\text{decopm}},{\text{SM}}_{n}^{\text{lefto}}\right\}. \end{array}$$

Furthermore, the decomposed signals SM_*n*_^decomp^ are processed by an optimized DCN for denoising the signals, which are forwarded to inverse EMCD to recover the restored source signals. Hence, the retrieved signals are obtained by adding the restored denoised signals with the leftover signals.

### 4.3. Prevention of Ocular Artifacts by Optimized DCN

The prevention or mitigation phase of ocular artifacts uses an optimized DCN for removing the noise from the given input signals. The decomposed signals from the EMCD are further given as input to optimized DCN to denoise the signals. The optimized DCN is trained by assigning the input as decomposed signals SM_*n*_^decomp^ and the target as denoised signals SM_*n*_^dnoise^. This trained optimized DCN by DS-EFO performs the signal denoising process in an efficient manner. The denoised signals are further given to the inverse EMCD to attain the restored denoised signals SM_*n*_^rede^. The leftover signal SM_*n*_^lefto^ and the restored denoised signals SM_*n*_^rede^ are concatenated to produce the artifacts removed signal or retrieved signal *SM*_*op*_^*retrv*^, which is the output signal of the mitigation phase without any artifacts. The equation is denoted in Eq. (37).

(37) $$\begin{array}{c} {\text{SM}}_{\text{op}}^{\text{retrv}}=\text{S}{\text{M}}_{n}^{\text{rede}}+.{\text{SM}}_{n}^{\text{lefto}} \end{array}$$

### 4.4. Objective Model for Detection and Prevention

The proposed ocular artifact removal model consists of two phases such as detection and prevention of ocular artifacts from EEG signals. The efficiency of the proposed model is verified by validating the multiobjective function.

Detection phase: although the DCN performs efficiently in the detection process of artifacts, it has some limitations in terms of accuracy when deal with a large number of training datasets. Therefore, in the proposed model, the epoch *E*_ch_ and learning rate Le_rt_ of DCN are optimized using DS-EFO, which is in the range of 10 to 20 and 0.1 to 0.9, respectively. The main objective of optimized DCN is to improve the classification or detection process concerning maximization of accuracy (accy) and precision (prcn).

(38) $$\begin{array}{c} \begin{array}{c} \text{fr }1=\underset{\left\{{\text{E}}_{\text{ch}},\text{L}{\text{e}}_{\text{rt}}\right\}}{\arg}\min\left(\frac{1}{\text{accy}+\text{prcn}}\right). \end{array} \end{array}$$

Accuracy accy is referred as “the nearness of the measurements to a specific value.” It is formulated in Eq. (39).

(39) $$\begin{array}{c} \text{accr}=\frac{\left({\text{ta}}_{p}+{\text{ta}}_{n}\right)}{\left({\text{ta}}_{p}+{\text{ta}}_{n}+{\text{fa}}_{p}+{\text{fa}}_{n}\right)}. \end{array}$$

Here, the term ta_*p*_ is denoted as true positives, fa_*p*_ is denoted as false positives, ta_*n*_ is denoted as true negatives, and fa_*n*_ is denoted as false negatives. Precision prcn is referred as “the points that are stated to be positive especially it is used to declare what percentage of the points is truly positive” as denoted in Eq. (40).

(40) $$\begin{array}{c} \text{prcn}=\frac{{\text{ta}}_{p}}{{\text{ta}}_{p}+\text{f}{\text{a}}_{p}}. \end{array}$$

Therefore, the efficiency of the ocular artifacts detection is enhanced by the optimized DCN by DS-EFO.

Prevention phase: the ocular artifact removal models use DCN for denoising the decomposed signals. The efficiency of DCN is improved by optimizing the DCN parameters by DS-EFO. The objective function of the optimized DCN removing ocular artifacts from EEG signals is the minimization of MAE between the clean signal and the artifacts removed signal or retrieved signal. The MAE metric “compares with the artifact reduction method ability to represent artifact waveforms because it provides an intuitive interpretation of the reconstruction errors by remaining their original units.” The equation of MAE is denoted in Eq. (41).

(41) $$\begin{array}{c} \text{MAE}=\frac{1}{\text{NF}}\overset{\text{NF}}{\underset{\text{ne}=1}{\sum }}\left|{\text{SM}}_{\text{op}}^{\text{retrv}}‐\text{S}{\text{M}}_{n}^{\text{cln}}\right|. \end{array}$$

Here, the time index is denoted as ne, and the term NF denotes the time points. The term SM_*n*_^cln^ indicates the clean signal. Thus, the objective function for the optimized DCN is given in Eq. (42).

(42) $$\begin{array}{c} \text{fr}2=\underset{\left\{{\text{E}}_{\text{ch}},\text{L}{\text{e}}_{\text{rt}}\right\}}{\text{argmin}}\left(\text{MAE}\right). \end{array}$$

Therefore, the proposed detection and removal model of ocular artifacts from EEG signals provide enhanced performance by optimizing the epoch and learning rates of DCN by DS-EFO algorithm. In the detection phase, the optimized DCN efficiently classifies the ocular artifacts by signals with artifacts and signals without artifacts. In the prevention phase, the input signals are denoised efficiently using the optimized DCN.

## 5. Results and Discussion

### 5.1. Experimental Setup

The proposed model for the detection and mitigation of ocular artifacts from EEG signals was implemented using MATLAB 2020a, and the performance evaluation was conducted by following measures. The dataset used for validating the proposed model was collected from (URL: http://www.bbci.de/competition/iv/#datasets, Access date: 2021-06-22) (Table 1). The experimental analysis was conducted by considering 9 subjects and the population size as 10, and the number of iterations performed as 100. The detection phase of the proposed DS-DFO-DCN was compared over the existing heuristic algorithms such as particle swarm optimization (PSO) [8], grey wolf optimization (GWO) [7], dual positioned elitism-based earth worm optimization algorithm-DCN (DPE-EWA-DCN) [38], and EFO [11] classifiers such as neural networks [15], SVM [12], EMCD+DPE-EWA-LWT [38], and DCN [7].

### 5.2. Performance Measures

Various performance metrics are considered for evaluating the performance of detection and prevention of ocular artifacts model that are given below:

(a) Sensitivity: it measures “the number of true positives, which are recognized exactly”
  (43) $$\begin{array}{c} \text{Sen}=\frac{{\text{ta}}_{p}}{{\text{ta}}_{p}+{\text{fa}}_{n}}. \end{array}$$
(b) Specificity: it measures “the number of true negatives, which are determined precisely”
  (44) $$\begin{array}{c} \text{Spe}=\frac{{\text{ta}}_{n}}{{\text{fa}}_{p}} \end{array}$$
(c) FPR: it is computed as “the ratio of the count of false positive predictions to the entire count of negative predictions”
  (45) $$\begin{array}{c} \text{FPR}=\frac{{\text{fa}}_{p}}{{\text{fa}}_{p}+{\text{ta}}_{n}}. \end{array}$$
(d) FNR: it is “the proportion of positives which yield negative test outcomes on the test”
  (46) $$\begin{array}{c} \text{FNR}=\frac{{\text{fa}}_{n}}{{\text{ta}}_{n}+{\text{ta}}_{p}} \end{array}$$
(e) NPV: it is the “probability that subjects with a negative screening test truly don't have the disease”
  (47) $$\begin{array}{c} \text{NPV}=\frac{{\text{fa}}_{n}}{{\text{fa}}_{n}+{\text{ta}}_{n}}. \end{array}$$
(f) FDR: it is “the number of false positives in all of the rejected hypotheses”
  (48) $$\begin{array}{c} \text{FDR}=\frac{{\text{fa}}_{p}}{{\text{fa}}_{p}+{\text{ta}}_{p}}. \end{array}$$
(g) F1 score: It is defined as the “harmonic mean between precision and recall. It is used as a statistical measure to rate performance”
  (49) $$\begin{array}{c} \text{F}1\text{ score}=\frac{2{\text{ta}}_{p}}{\left(2{\left(\text{ta}\right.}_{p}+{\text{fa}}_{p}+{\text{fa}}_{n}\right)}. \end{array}$$
(h) MCC: it is a “correlation coefficient computed by four values”
  (50) $$\begin{array}{c} \text{MCC}=\frac{{\text{ta}}_{p}\times {\text{ta}}_{n}-{\text{fa}}_{p}\times {\text{fa}}_{n}}{\sqrt{\left({\text{ta}}_{p}+{\text{fa}}_{p}\right)\left({\text{ta}}_{p}+{\text{fa}}_{n}\right)\left({\text{ta}}_{n}+{\text{fa}}_{p}\right)\left({\text{ta}}_{n}+{\text{fa}}_{n}\right)}}. \end{array}$$
(i) Correlation coefficient: it **“**considers the relative movements of the signals and then defines if there is any relationship between them”
  (51) $$\begin{array}{c} \text{corr}=\frac{n\left(\sum {\text{SM}}_{\text{op}}^{\text{retrv}}{\text{SM}}_{n}^{\text{cln}}\right)-\sum \left({\text{SM}}_{\text{op}}^{\text{retrv}}\right)\left({\text{SM}}_{n}^{c\ln}\right)}{\sqrt{\begin{array}{c} \left[n\sum {\left({\text{SM}}_{\text{op}}^{\text{retrv}}\right)}^{2}-{\left(\sum \left({\text{SM}}_{\text{op}}^{\text{retrv}}\right)\right)}^{2}\right]\\ \left[n\sum {\left({\text{SM}}_{n}^{c\ln}\right)}^{2}-{\left(\sum {\text{SM}}_{n}^{c\ln}\right)}^{2}\right] \end{array}}}. \end{array}$$
(j) RMSE: RMSE “is a quadratic scoring rule that measures the average magnitude of the error. It's the square root of the average of the squared differences between prediction and actual observations”
  (52) $$\begin{array}{c} \text{RMSE}=\sqrt{\frac{1}{\text{NF}}\overset{\text{NF}}{\underset{\text{ne}=1}{\sum }}{\left(\left|{\text{SM}}_{\text{op}}^{\text{retrv}}-{\text{SM}}_{n}^{c\ln}\right|\right)}^{2}}. \end{array}$$

### 5.3. Performance Analysis on MAE

**Figure 5:**
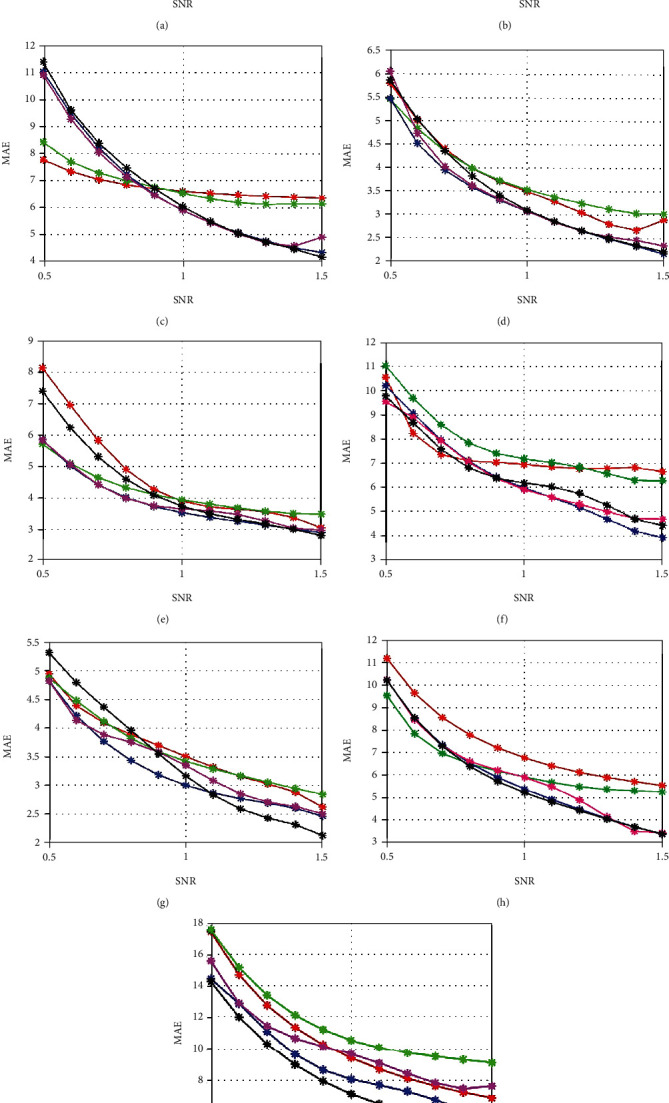
Analysis of the proposed DS-EFO-DCN detection and mitigation of ocular artifacts with existing metaheuristic algorithms in terms of MAE for (a) subject 1, (b) subject 2, (c) subject 3, (d) subject 4, (e) subject 5, (f) subject 6, (g) subject 7, (h) subject 8, and (i) subject 9.

The performance analysis of the proposed ocular artifact detection and mitigation model on MAE is evaluated between the retrieved signal and clean signal in semisimulated data generation. The proposed DS-EFO-DCN is compared with other heuristic algorithms in terms of MAE with 9 subjects that are depicted in Figure 5. The proposed DS-EFO-DCN achieves minimum error rates when gradually increasing the SNR rate from 0.5 to 1.5. The EFO-DCN and the DPE-EWA-DCN attained more or less similar MAE rates like the proposed DS-EFO-DCN while varying the SNR rate for all 9 subjects when compared with the PSO and GWO algorithms. In subject 2, the MAE of proposed DS-EFO-DCN for SNR value 1.5 is 46.15% better than PSO-DCN, 40% better than GWO-DCN, 16% better than DPE-EWA-DCN, and 27.59% better than EFO-DCN. Likewise, for all subjects, the proposed DS-EFO-DCN attains minimum MAE values when compared with conventional algorithms for the prevention of ocular artifacts.

### 5.4. Performance Analysis on RMSE

The performance of the DS-EFO-DCN is compared with the other heuristic algorithms by evaluating the RMSE value for all 9 subjects as shown in Table 2. The minimum error rate value was attained by the proposed DS-EFO-DCN while increasing the SNR rate from the range of 0.5 to 1.5 when compared with the conventional algorithms. In all 9 subjects, the EFO-DCN and the DPE-EWA-DCN also reach more or less same RMSE rate while varying the SNR rate like the proposed DS-EFO-DCN when compared with the PSO and GWO algorithms. In subject 9, the RMSE of proposed DS-EFO-DCN for SNR value 1 is 40% better than PSO-DCN, 50% better than GWO-DCN, 23.07% better than DPE-EWA-DCN, and 45.45% better than EFO-DCN. Similarly, the proposed DS-EFO-DCN attains minimum RSME values for the remaining subjects when compared with conventional algorithms for the prevention of ocular artifacts.

Again, performance analysis on RMSE has evaluated through chirplet transform-based time-frequency images of both 5 s and 8 sec frames of EEG signals. The resultant is presented in Table 3. It is evident that the time-frequency images of 5 s and 8 s EEG frames coupled with the proposed scheme obtained average accuracy value of 97.66% and 96.65%, respectively. These accuracy values are comparatively good as compared to other existing method.

### 5.5. Performance Analysis on Correlation Coefficient

**Figure 6:**
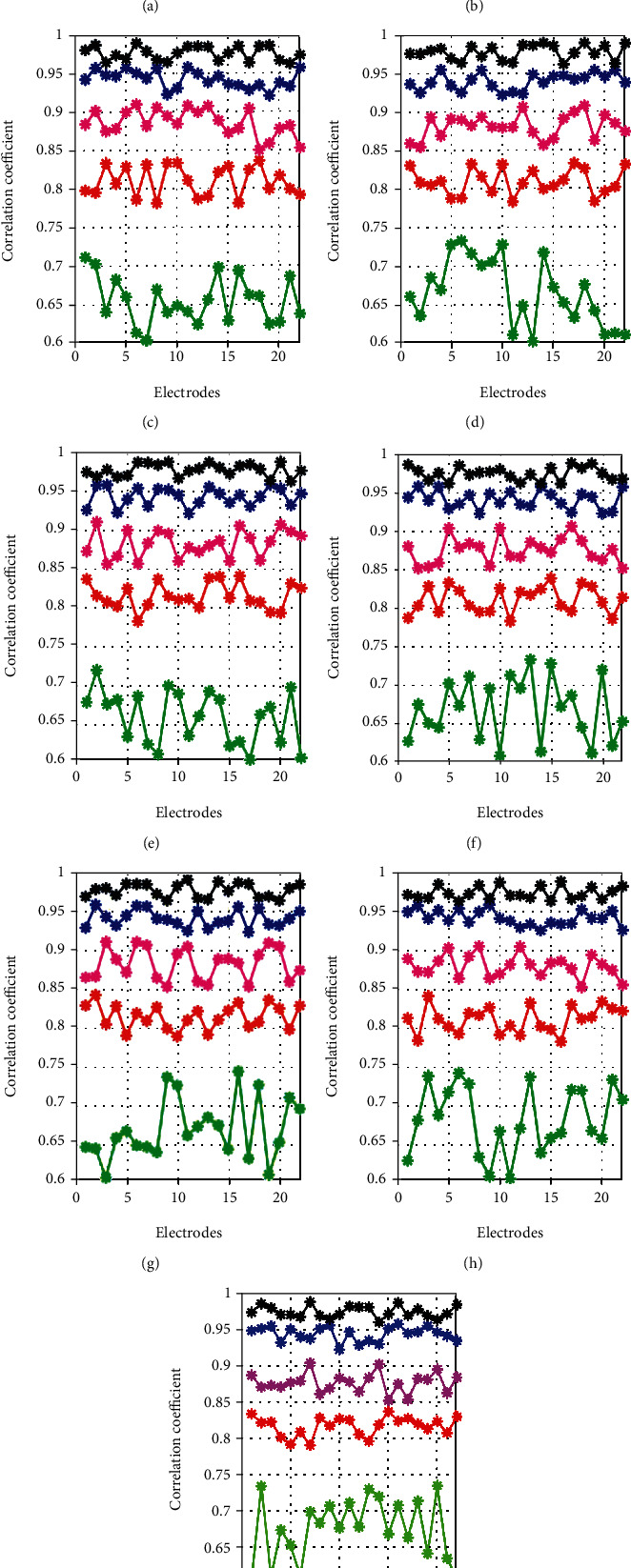
Analsyis of mitigation of ocular artifacts of the proposed DS-EFO-DCN model with metaheuristic algorithms in terms of correlation coefficient for (a) subject 1, (b) subject 2, (c) subject 3, (d) subject 4, (e) subject 5, (f) subject 6, (g) subject 7, (h) subject 8, and (i) subject 9.

The correlation coefficient analysis on the performance of the DS-EFO-DCN for all 9 subjects is compared with the other heuristic algorithms as shown in Figure 6. The high correlation efficient rate was attained by the proposed DS-EFO-DCN for the selected electrodes when compared with the conventional algorithms. In all 9 subjects, the EFO-DCN and the DPE-EWA-DCN attained more or less the same correlation coefficient rate while taking different electrodes DS-EFO-DCN, whereas the PSO and GWO algorithms attained very less value of correlation coefficient. In subject 5, the correlation coefficient of proposed DS-EFO-DCN for electrode no. 5 is 16.87% better than PSO-DCN, 58% better than GWO-DCN, 3.19% better than DPE-EWA-DCN, and 7.77% better than EFO-DCN. In the same way, the proposed DS-EFO-DCN attains high correlation values for the remaining subjects in mitigating the artifacts when compared with existing algorithms.

### 5.6. Performance Analysis on Correlation Coefficient

The semisimulated data generation is used to validate the mitigation of ocular artifacts, since there is no proof to validate the measures. Therefore, the difference between the denoised and the clean signal is validated by autocorrelation evaluation. While processing the evaluation, there should not be any data loss other than artifacts. Thus, the best performance is attained by reaching a high correlation coefficient value. Correlation coefficient of the proposed DS-EFO-DCN is attained maximum rate when compared with the conventional algorithms. The proposed DS-EFO-DCN is compared with other conventional algorithms with 22 numbers of electrodes for each subject, and the results with regards to the correlation coefficient for all 9 subjects are represented in Table 4. From Table 4, while taking electrode 5 of subject 2, the performance of the proposed DS-EFO-DCN is 15.22% better than PSO-DCN, 44.92% better than GWO-DCN, 1.46% better than DPE-EWA-DCN, and 12.29% better than EFO-DCN. Therefore, the proposed approach has attained a high correlation coefficient, which improves the prevention strategy when compared with other algorithms.

### 5.7. Overall Performance Analysis of Detection

The performance of the proposed DS-EFO-DCN detection and mitigation model is analyzed with the existing metaheuristic algorithms and is represented in Table 5. The accuracy of the proposed DS-EFO-DCN model is 4.42% better than PSO-DCN, 0.82% better than GWO-DCN, 2.67% better than DPE-EWA-DCN, and 3.02% better than EFO-DCN. Similarly, the proposed model attains better performance for all performance metrics. In the same way, the performance analysis of the proposed DS-EFO-DCN model with the existing classifiers is represented in Table 6.

The precision of the proposed model is 24.44% better than NN, 5.97% better than SVM, 27% better than DPE-EWA-DCN, and 15.35% better than DCN. Therefore, the overall analysis reveals that the proposed DS-EFO-DCN algorithm of detection and mitigation model provides better performance than the existing algorithms.

The computational complexity with simulation time of proposed scheme was also compared with the existing methods of artifact elimination approach. The comparison outcomes are presented in Table 7. Here, the EEG signal's length (*E*_l_) for comparing computational complexities of different artifact removal approach is selected as 38000 samples. The proposed scheme has been implemented in MATLAB-2019a version software along with 64-bit personal computer of 10GB RAM and Intel 5 core i3 processor at 2.476Ghz. The proposed method has a simulation time value of 2.89 second, which is optimum interval as compared to other existing scheme by considering ICA as a base line. Hence, the proposed scheme is computationally feasible and has better denoising performance for artifacts removal from EEG signal.

## 6. Conclusion

A new approach for the detection and mitigation of ocular artifacts from EEG signals was introduced in this proposed research work. The projected model has two phases such as detection phase and mitigation phase. In the detection part, the input EEG signals were decomposed through 5-level DWT and Pisarenko harmonic decomposition techniques. The features of decomposed signals were extracted by PCA and ICA. Then, the extracted features were given to the optimized DCN, in which the optimization was done by DS-EFO algorithm. The optimized DCN classifies the signals into signal with artifacts and without artifacts. In the mitigation part, the semisimulated data was generated for validating the detection of artifacts. Here, the moderation of ocular artifacts from EEG signals was done by the same optimized DCN using the proposed DS-EFO. The performance analysis of the proposed DS-EFO-DCN algorithm ensures the enhanced results over the existing metaheuristic algorithms in terms of MAE, RMSE, and correlation coefficients. From the overall analysis, the specificity evaluation of the proposed DS-EFO-DCN model has achieved 5.03% better than NN, 0.93% better than SVM, 2.84% better than EMCD+DPE-EWA-LWT, and 3.23% better than DCN. Thus, it is concluded that the developed DS-EFO-DCN model achieves better performance in the detection and mitigation of ocular artifacts from EEG signals.

## Figures and Tables

**Algorithm 1:**
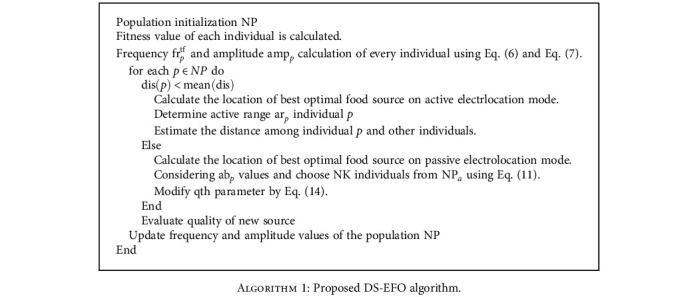
Proposed DS-EFO algorithm.

**Table 1:** EEG episodes used in this proposed work for detection and mitigation of ocular artifacts.

Database	EEG Channel	EOG Channel	Total
BCI competition IV database	22	03	25 channel signals with 288 epochs

**Table 2:** Analysis of proposed DS-EFO-DCN ocular artifacts detection and mitigation model over existing meta heuristic algorithms in terms of RMSE for (A) Subject 1, (B) Subject 2, (C) Subject 3, (D) Subject 4, (E) Subject 5, (F) Subject 6, (G) Subject 7, (H) Subject 8, and (I) Subject 9.

Subject No.	SNR	RMSE				
		PSO-DCN [8]	GWO-DCN [7]	DPE-EWA-DCN [38]	EFO-DCN [11]	DS-EFO-DCN
Subject 1	0.5	6.1	7.0	7.8	9.1	8.8
	0.7	5.5	6.9	6.8	6.7	6.5
	1.0	4.1	6.3	5.5	5.0	4.5
	1.3	3.8	5.5	5.2	5.0	4.0
	1.5	3.6	5.1	5.0	5.1	3.2

Subject 2	0.5	4.9	5.0	5.8	6.5	6.5
	0.7	4.4	4.3	4.7	4.5	4.5
	1.0	3.8	3.9	3.4	3.4	3.3
	1.3	3.5	3.7	2.8	2.8	2.5
	1.5	3.8	3.5	2.4	2.7	2.1

Subject 3	0.5	7.8	8.4	11.0	10.9	11.1
	0.7	7.0	7.3	8.0	8.0	8.3
	1.0	6.8	6.8	6.0	5.9	6.0
	1.3	6.5	6.2	4.8	4.7	4.6
	1.5	6.2	6.0	4.3	5.0	4.0

Subject 4	0.5	5.7	5.5	5.5	6.0	5.7
	0.7	4.4	4.4	3.8	3.6	4.4
	1.0	3.5	3.5	3.0	3.0	3.0
	1.3	2.7	3.1	2.5	2.6	2.5
	1.5	2.7	3.0	2.3	2.4	2.2

Subject 5	0.5	8.0	5.8	5.8	5.8	7.1
	0.7	5.8	4.4	4.5	4.5	5.2
	1.0	4.0	4.0	3.5	3.6	3.8
	1.3	3.7	3.8	3.2	3.3	3.2
	1.5	3.0	3.5	2.8	2.9	2.8

Subject 6	0.5	10.5	11.0	10.1	10.6	9.8
	0.7	7.3	8.8	8.0	8.0	7.7
	1.0	7.0	7.3	6.0	6.0	6.0
	1.3	6.9	6.7	4.8	5.0	5.1
	1.5	6.7	6.3	4.0	4.9	4.5

Subject 7	0.5	5.0	4.8	4.7	4.7	5.4
	0.7	4.2	4.3	3.7	3.8	4.4
	1.0	3.5	3.4	3.0	3.4	3.2
	1.3	3.1	3.1	2.7	2.7	2.4
	1.5	2.6	2.7	2.5	2.6	2.1

Subject 8	0.5	11.0	9.7	10.1	10.1	10.0
	0.7	8.7	7.0	7.3	7.3	7.2
	1.0	6.8	5.9	5.2	6.0	5.1
	1.3	5.9	5.5	4.2	4.3	4.0
	1.5	5.5	5.5	3.4	3.5	3.4

Subject 9	0.5	17.8	17.9	14.2	15.8	14.0
	0.7	12.5	13.8	13.5	11.7	10.3
	1.0	9.8	10.5	8.0	9.9	7.3
	1.3	7.9	9.8	7.7	8.0	5.8
	1.5	7.5	9.5	6.0	7.8	4.5

**Table 3:** Classification using time-frequency domain analysis.

Window size	Accuracy (%)	Sensitivity	Specificity	F1-score (%)	MCC (%)
5 s	97.66 ± 1.52	98.54 ± 1.08	94.66 ± 3.17	97.36 ± 1.67	94.56 ± 2.87
8 s	96.65 ± 2.07	97.94 ± 3.73	93.85 ± 3.67	96.75 ± 1.87	93.55 ± 3.77

**Table 4:** Performance analysis after removing ocular artifacts without semisimulated data generation in terms of correlation coefficient for all 9 subjects.

Electrodes	PSO-DCN [8]	GWO-DCN [7]	DPE-EWA-DCN [38]	EFO-DCN [11]	DS-EFO-DCN	Electrodes	PSO-DCN [8]	GWO-DCN [7]	DPE-EWA-DCN [38]	EFO-DCN [11]	DS-EFO-DCN

Subject 1						Subject 2					

1	0.810	0.652	0.953	0.860	0.961	1	0.8029	0.719	0.922	0.875	0.978
2	0.802	0.715	0.923	0.901	0.976	2	0.811	0.659	0.953	0.850	0.984
3	0.824	0.624	0.937	0.885	0.965	3	0.833	0.697	0.928	0.886	0.962
4	0.811	0.618	0.921	0.872	0.981	4	0.830	0.687	0.952	0.907	0.988
5	0.828	0.723	0.948	0.8633	0.975	5	0.833	0.663	0.947	0.855	0.960
6	0.829	0.606	0.946	0.863	0.967	6	0.836	0.666	0.953	0.852	0.984
7	0.791	0.696	0.928	0.881	0.986	7	0.828	0.732	0.955	0.903	0.984
8	0.787	0.702	0.945	0.876	0.981	8	0.780	0.611	0.925	0.864	0.961
9	0.829	0.661	0.941	0.894	0.985	9	0.780	0.639	0.934	0.850	0.975
10	0.818	0.653	0.952	0.854	0.962	10	0.785	0.662	0.957	0.898	0.987
11	0.780	0.737	0.941	0.901	0.965	11	0.795	0.682	0.944	0.858	0.969
12	0.833	0.656	0.954	0.890	0.981	12	0.781	0.722	0.954	0.902	0.983
13	0.810	0.663	0.935	0.858	0.980	13	0.805	0.665	0.938	0.855	0.977
14	0.812	0.621	0.933	0.901	0.982	14	0.800	0.661	0.920	0.871	0.989
15	0.816	0.644	0.938	0.861	0.974	15	0.812	0.704	0.943	0.885	0.983
16	0.825	0.643	0.929	0.886	0.972	16	0.835	0.665	0.928	0.885	0.961
17	0.831	0.725	0.955	0.882	0.977	17	0.797	0.720	0.940	0.890	0.968
18	0.802	0.634	0.937	0.859	0.984	18	0.800	0.665	0.947	0.888	0.978
19	0.785	0.643	0.929	0.850	0.979	19	0.831	0.669	0.943	0.876	0.974
20	0.824	0.657	0.936	0.896	0.975	20	0.800	0.668	0.942	0.858	0.988
21	0.799	0.699	0.946	0.895	0.968	21	0.788	0.632	0.936	0.895	0.981
22	0.830	0.620	0.935	0.875	0.980	22	0.810	0.618	0.929	0.864	0.976

Subject 3						Subject 4					

Electrodes	PSO-DCN [8]	GWO-DCN [7]	DPE-EWA-DCN [38]	EFO-DCN [11]	DS-EFO-DCN	Electrodes	PSO-DCN [8]	GWO-DCN [7]	DPE-EWA-DCN [38]	EFO-DCN [11]	DS-EFO-DCN

1	0.797	0.71	0.941	0.883	0.979	1	0.830	0.659	80.936	0.858	0.975
2	0.795	0.701	0.956	0.901	0.986	2	0.808	0.634	0.925	0.854	0.974
3	0.833	0.639	0.947	0.874	0.963	3	0.805	0.684	0.937	0.893	0.979
4	0.806	0.681	0.946	0.878	0.973	4	0.810	0.667	0.954	0.868	0.982
5	0.828	0.658	0.956	0.900	0.968	5	0.788	0.726	0.933	0.890	0.969
6	0.785	0.612	0.949	0.909	0.989	6	0.788	0.731	0.925	0.889	0.963
7	0.831	0.603	0.943	0.881	0.978	7	0.832	0.715	0.942	0.882	0.984
8	0.781	0.669	0.956	0.906	0.967	8	0.816	0.699	0.953	0.893	0.971
9	0.833	0.639	0.922	0.894	0.964	9	0.796	0.704	0.933	0.880	0.982
10	0.834	0.648	0.930	0.884	0.976	10	0.832	0.726	0.922	0.879	0.965
11	0.811	0.640	0.957	0.908	0.984	11	0.783	0.609	0.925	0.880	0.963
12	0.787	0.624	0.949	0.899	0.984	12	0.807	0.647	0.923	0.906	0.986
13	0.79	0.656	0.938	0.908	0.984	13	0.823	0.601	0.948	0.873	0.985
14	0.822	0.698	0.946	0.889	0.966	14	0.800	0.716	0.937	0.857	0.989
15	0.829	0.629	0.936	0.873	0.976	15	0.804	0.671	0.945	0.864	0.985
16	0.782	0.693	0.934	0.879	0.985	16	0.812	0.651	0.947	0.891	0.961
17	0.825	0.662	0.928	0.905	0.964	17	0.834	0.632	0.942	0.900	0.976
18	0.837	0.661	0.935	0.851	0.985	18	0.827	0.675	0.944	0.908	0.989
19	0.8	0.625	0.921	0.859	0.986	19	0.784	0.641	0.953	0.863	0.975
20	0.818	0.627	0.938	0.878	0.966	20	0.797	0.610	0.945	0.896	0.985
21	0.8	0.686	0.933	0.883	0.962	21	0.803	0.612	0.953	0.885	0.962
22	0.792	0.638	0.957	0.854	0.974	22	0.832	0.610	0.938	0.874	0.989

Subject 5						Subject 6					

Electrodes	PSO-DCN [8]	GWO-DCN [7]	DPE-EWA-DCN [38]	EFO-DCN [11]	DS-EFO-DCN	Electrodes	PSO-DCN [8]	GWO-DCN [7]	DPE-EWA-DCN [38]	EFO-DCN [11]	DS-EFO-DCN

1	0.835	0.674	0.925	0.871	0.974	1	0.787	0.626	0.944	0.880	0.985
2	0.814	0.716	0.956	0.909	0.968	2	0.803	0.674	0.958	0.851	0.978
3	0.806	0.672	0.957	0.855	0.977	3	0.829	0.650	0.940	0.853	0.966
4	0.800	0.677	0.921	0.865	0.967	4	0.795	0.644	0.958	0.858	0.975
5	0.823	0.630	0.939	0.899	0.969	5	0.833	0.702	0.929	0.904	0.962
6	0.781	0.682	0.953	0.855	0.986	6	0.823	0.672	0.935	0.878	0.985
7	0.802	0.620	0.929	0.882	0.986	7	0.803	0.711	0.946	0.884	0.973
8	0.835	0.607	0.952	0.898	0.983	8	0.795	0.629	0.922	0.880	0.976
9	0.813	0.696	0.951	0.894	0.987	9	0.795	0.695	0.948	0.854	0.976
10	0.808	0.685	0.944	0.859	0.965	10	0.826	0.607	0.936	0.904	0.980
11	0.810	0.631	0.920	0.876	0.975	11	0.783	0.712	0.951	0.867	0.971
12	0.799	0.657	0.934	0.871	0.978	12	0.821	0.695	0.934	0.866	0.962
13	0.837	0.688	0.954	0.879	0.986	13	0.817	0.732	0.932	0.886	0.973
14	0.839	0.678	0.946	0.885	0.979	14	0.825	0.613	0.957	0.879	0.961
15	0.811	0.618	0.934	0.859	0.971	15	0.839	0.727	0.947	0.872	0.981
16	0.840	0.624	0.944	0.904	0.981	16	0.803	0.671	0.936	0.889	0.961
17	0.807	0.600	0.929	0.888	0.984	17	0.796	0.686	0.924	0.906	0.988
18	0.806	0.659	0.942	0.860	0.977	18	0.833	0.644	0.948	0.887	0.982
19	0.793	0.668	0.957	0.884	0.962	19	0.828	0.611	0.944	0.867	0.987
20	0.792	0.622	0.952	0.906	0.987	20	0.808	0.719	0.923	0.862	0.975
21	0.830	0.693	0.931	0.897	0.962	21	0.830	0.693	0.931	0.897	0.962
22	0.824	0.603	0.946	0.891	0.975	22	0.824	0.603	0.946	0.891	0.975

Subject 7						Subject 8					

Electrodes	PSO-DCN [8]	GWO-DCN [7]	DPE-EWA-DCN [38]	EFO-DCN [11]	DS-EFO-DCN	Electrodes	PSO-DCN [8]	GWO-DCN [7]	DPE-EWA-DCN [38]	EFO-DCN [11]	DS-EFO-DCN

1	0.826	0.641	0.926	0.862	0.967	1	0.810	0.624	0.948	0.887	0.970
2	0.840	0.639	0.956	0.863	0.976	2	0.781	0.677	0.955	0.870	0.966
3	0.801	0.601	0.941	0.909	0.978	3	0.839	0.738	0.939	0.870	0.965
4	0.825	0.652	0.930	0.886	0.969	4	0.810	0.684	0.950	0.884	0.983
5	0.787	0.661	0.942	0.868	0.983	5	0.799	0.712	0.937	0.901	0.971
6	0.816	0.643	0.955	0.908	0.983	6	0.790	0.733	0.952	0.861	0.961
7	0.806	0.641	0.954	0.904	0.983	7	0.817	0.723	0.934	0.890	0.970
8	0.824	0.634	0.938	0.861	0.970	8	0.814	0.624	0.948	0.904	0.982
9	0.796	0.731	0.937	0.850	0.962	9	0.825	0.605	0.9571	0.861	0.964
10	0.786	0.720	0.932	0.893	0.981	10	0.789	0.666	0.939	0.869	0.986
11	0.807	0.656	0.922	0.902	0.989	11	0.801	0.603	0.936	0.879	0.969
12	0.818	0.667	0.949	0.857	0.965	12	0.784	0.662	0.927	0.903	0.969
13	0.788	0.679	0.925	0.852	0.963	13	0.834	0.737	0.932	0.880	0.965
14	0.807	0.669	0.934	0.886	0.986	14	0.807	0.635	0.924	0.866	0.982
15	0.819	0.638	0.935	0.886	0.974	15	0.791	0.651	0.934	0.882	0.962
16	0.830	0.739	0.954	0.881	0.985	16	0.786	0.664	0.932	0.884	0.987
17	0.798	0.626	0.921	0.850	0.983	17	0.821	0.716	0.933	0.874	0.964
18	0.804	0.721	0.952	0.891	0.965	18	0.804	0.715	0.951	0.850	0.968
19	0.833	0.605	0.931	0.907	0.967	19	0.817	0.663	0.940	0.892	0.979
20	0.822	0.646	0.930	0.902	0.962	20	0.835	0.653	0.939	0.880	0.964
21	0.795	0.705	0.939	0.857	0.978	21	0.827	0.729	0.949	0.872	0.974
22	0.826	0.690	0.948	0.871	0.983	22	0.829	0.703	0.924	0.853	0.980

Subject 9												

Electrodes	PSO-DCN [8]	GWO-DCN [7]	DPE-EWA-DCN [38]	EFO-DCN [11]	DS-EFO-DCN	Electrodes	PSO-DCN [8]	GWO-DCN [7]	DPE-EWA-DCN [38]	EFO-DCN [11]	DS-EFO-DCN

1	0.835	0.609	0.948	0.888	0.973	12	0.808	0.680	0.928	0.865	0.980
2	0.823	0.735	0.951	0.871	0.987	13	0.798	0.732	0.935	0.884	0.980
3	0.824	0.613	0.955	0.874	0.979	14	0.821	0.722	0.930	0.902	0.960
4	0.804	0.676	0.932	0.872	0.970	15	0.839	0.671	0.951	0.853	0.970
5	0.794	0.656	0.950	0.878	0.970	16	0.824	0.710	0.957	0.876	0.986
6	0.811	0.614	0.940	0.880	0.967	17	0.829	0.666	0.944	0.855	0.969
7	0.793	0.701	0.937	0.904	0.987	18	0.822	0.716	0.946	0.883	0.977
8	0.830	0.685	0.951	0.862	0.968	19	0.815	0.645	0.955	0.882	0.968
9	0.819	0.709	0.956	0.870	0.964	20	0.825	0.736	0.946	0.896	0.963
10	0.828	0.679	0.922	0.884	0.970	21	0.809	0.638	0.941	0.863	0.971
11	0.827	0.713	0.946	0.879	0.981	22	0.831	0.610	0.934	0.885	0.983

**Table 5:** Overall performance analysis of the proposed DS-EFO-DCN detection and mitigation model with metaheuristic based algorithms.

Performance measures	PSO-DCN [8]	GWO-DCN [7]	DPE-EWA-DCN [38]	EFO-DCN [11]	DS-EFO-DCN
Accuracy (%)	90.40	93.60	91.90	91.60	94.40
Sensitivity (%)	92.10	92.10	90.70	90.70	92.10
Specificity (%)	90.20	93.80	92.10	91.70	94.70
Precision	55.5	66.6	60.5	59.4	70.1
FPR	9.7	6.1	7.8	8.2	5.2
FNR	7.8	7.8	9.2	9.2	7.8
NPV	90.2	93.8	92.1	91.7	94.7
FDR	44.4	33.3	39.4	40.5	30.1
F1-score	69.3	77.3	72.6	71.8	79.5
MCC	66.9	75.0	70.0	69.3	77.3

**Table 6:** Overall performance analysis of the proposed DS-EFO-DCN detection and mitigation model with existing classifiers.

Performance measures	NN [15]	SVM [12]	EMCD+DPE-EWA-LWT [38]	DCN [26]	DS-EFO-DCN
Accuracy (%)	90.7	93.6	90.2	92.1	94.4
Sensitivity (%)	94.7	94.7	92.1	93.4	92.1
Specificity (%)	90.2	93.5	90.0	91.9	94.7
Precision	56.20	66.00	55.10	60.60	70.10
FPR	9.70	6.40	9.90	8.00	5.20
FNR	5.20	5.20	7.80	6.50	7.80
NPV	90.20	93.50	90.10	91.90	94.70
FDR	43.70	33.90	44.80	39.30	30.10
F1-score	70.50	77.80	68.90	73.50	79.50
MCC	68.60	75.90	66.50	71.42	77.30

**Table 7:** Computational complexity comparison of various existing schemes with the proposed method.

Methods	Simulation time in second
5-level DWT and Pisarenko harmonic method [35]	12.86
EMCD-ICA [26]	25.75
PSO-DCN [8]	10.45
GWO-DCN [7]	48.52
DPE-EWA-DCN [38]	30.75
EFO-DCN [11]	75.12
Proposed DS-EFO-DCN	2.89

## Data Availability

The [URL: http://www.bbci.de/competition/iv/#datasets] data used to support the findings of this study are included within the article.
